# Fbxl8 suppresses lymphoma growth and hematopoietic transformation through degradation of cyclin D3

**DOI:** 10.1038/s41388-020-01532-4

**Published:** 2020-10-29

**Authors:** Akihiro Yoshida, Jaewoo Choi, Hong Ri Jin, Yan Li, Sagar Bajpai, Shuo Qie, J. Alan Diehl

**Affiliations:** 1 Department of Biochemistry, Case Comprehensive Cancer Center, Case Western Reserve University, Cleveland, OH 44106 USA; 2 Department of Biochemistry and Molecular Biology, Hollings Cancer Center, Medical University of South Carolina, Charleston, SC 29425 USA; 3 Case Comprehensive Cancer Center, Case Western Reserve University, Cleveland, OH 44106 USA; 4 Abramson Family Cancer Research Institute, Department of Cancer Biology, Abramson Cancer Center, University of Pennsylvania, Philadelphia, PA 19104 USA

**Keywords:** Ubiquitylation, Lymphoma

## Abstract

Overexpression of D-type cyclins in human cancer frequently occurs as a result of protein stabilization, emphasizing the importance of identification of the machinery that regulates their ubiqutin-dependent degradation. Cyclin D3 is overexpressed in ~50% of Burkitt’s lymphoma correlating with a mutation of Thr-283. However, the E3 ligase that regulates phosphorylated cyclin D3 and whether a stabilized, phosphorylation deficient mutant of cyclin D3, has oncogenic activity are undefined. We describe the identification of SCF-Fbxl8 as the E3 ligase for Thr-283 phosphorylated cyclin D3. SCF-Fbxl8 poly-ubiquitylates p-Thr-283 cyclin D3 targeting it to the proteasome. Functional investigation demonstrates that Fbxl8 antagonizes cell cycle progression, hematopoietic cell proliferation, and oncogene-induced transformation through degradation of cyclin D3, which is abolished by expression of cyclin D3T283A, a non-phosphorylatable mutant. Clinically, the expression of cyclin D3 is inversely correlated with the expression of Fbxl8 in lymphomas from human patients implicating Fbxl8 functions as a tumor suppressor.

## Highlights

- Fbxl8 suppresses cell division, cell proliferation, and tumorigenesis through phosphorylation-dependent degradation of cyclin D3.
- Fbxl8 suppresses oncogene-induced transformation of hematopoietic cells and lymphoma cell proliferation through cyclin D3 degradation.

## Introduction

Dysregulated expression of D type cyclins occurs frequently in human cancers including carcinomas of the esophagus and breast, as well numerous lymphoma subtypes such as mantle cell lymphoma [1–4], B cell lymphocytic leukemias [5], and Burkitt’s lymphomas [6]. Because expression of D cyclins is limiting, increased expression of individual D-type cyclins increases the catalytic activity of their catalytic partners, cyclin dependent kinases Cdk4 or Cdk6 leading to aberrant cell cycle progression and cell proliferation [7, 8]. While cyclin D1 is the primary D-type cyclin in fibroblasts, mammary epithelium and muscle cells, cyclins D2 and D3 are also expressed in these cell types at reduced levels and conversely are highly expressed in hematopoietic tissues [9]. While this expression pattern was thought to reflect gene expression, recent works on cyclin D1 proteolysis revealed that dysregulated ubiquitin-dependent degradation results in its aberrant expression in many tissues including B and T lymphocytes highlighting the importance of post-translational regulation [10, 11]. The frequent dysregulation in cancer juxtaposed with exquisite regulation of individual D-type cyclin accumulation in specific tissues underscores the importance of elucidating the molecular mechanisms that determine cyclin accumulation.

Mutation of Thr-286, in cyclin D1, to alanine generates a highly stable and constitutively nuclear cyclin D1 allele. This residue is frequently mutated in human cancers and this mutant allele has overt oncogenic activity in vitro and in mouse models [10, 12]. The mechanisms of tumor promotion by cyclin D1T286A, pertain to novel nuclear functions specifically during S-phase [12–14]. Cyclins D2 and D3 retain a conserved Thr analogous to cyclin D1-Thr-286 (Thr280 for cyclin D2 and Thr283 for cyclin D3), suggesting the potential for phosphorylation-dependent regulation of both. However, this possibility has not been established.

Evidence for the clinical relevance of post-translational regulation of cyclins D2 and D3 has been lacking. However, recent work revealed that cyclin D3 is mutated at Thr283 in 50% of Burkitt’s lymphomas supporting the importance of post-translational regulation of cyclin D3 with this residue Thr283 in lymphoma [6]. Finally, cyclin D3 has functions in lymphocyte development that cannot be rescued by other cyclins [15] highlighting the importance of elucidating the mechanisms that govern cyclin D3 post-translational regulation. Here we describe the identification and characterization of Fbxl8, an F-box protein with specificity for phosphorylated cyclin D3. Fbxl8 has not previously been shown to function as an E3 ligase. As described below, Fbxl8, directly polyubiquitylates cyclins D3 in a phosphorylation-dependent manner in vivo and in vitro thereby determining the rate of degradation. Loss of Fbxl8 results in cyclin D3 accumulation, cell cycle dysregulation and oncogene-driven transformation revealing tumor suppressor potential.

## Results

### Fbxl8 co-precipitates with cyclin D3, but not cyclins D1 and D2

There is a paucity of data characterizing cyclin D3 phosphorylation in vivo. Therefore, we immunopurified Flag-cyclin D3 from NIH3T3 cells, and using mass spectrometry analysis identified phosphorylation of 8 distinct serine/threonine residues including Thr-283 (Fig. S1A). Mutation of individual residues to alanine demonstrated that only a Thr-283 to alanine mutation (T283A) altered the kinetics of cyclin D3 degradation (Fig. S1B).

**Fig. 1:**
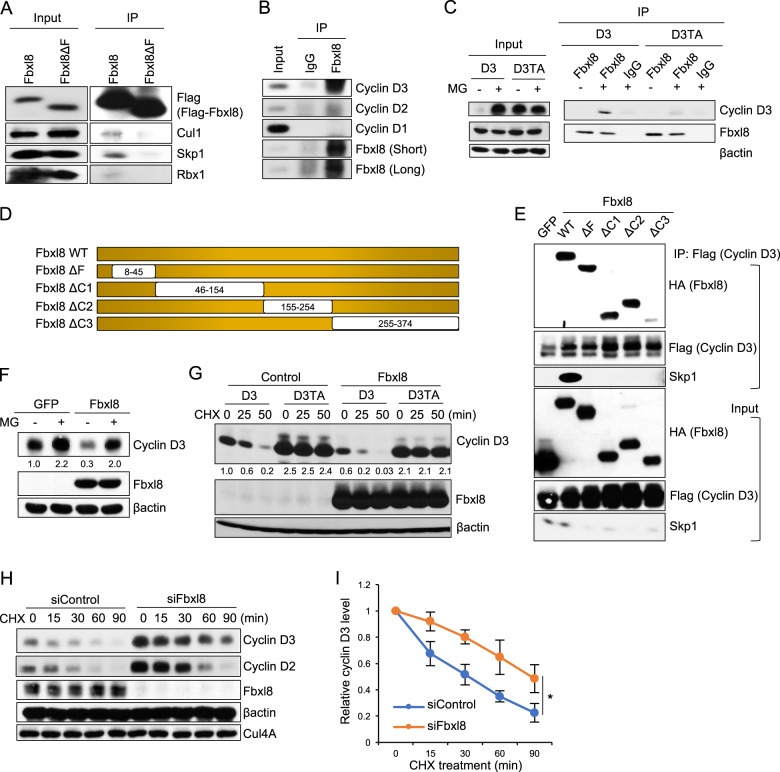
Fbxl8 binds to and regulates cyclin D3 in proteasome and phosphorylation-dependent manners. **a** Lysates from NIH3T3 cells transfected with Flag-Fbxl8 or Flag-Fbxl8ΔF and treated with a proteasome inhibitor MG132 (20 μM) for 4 h were immunoprecipitated with anti-Flag beads. Immune complexes were analyzed by western blot for Flag-Fbxl8, Cul1, Skp1, and Rbx1. **b** Lysates from NIH3T3 cells treated with a proteasome inhibitor MG132 (20 μM) for 4 h were immunoprecipitated with normal IgG or an antibody specific for Fbxl8. Immune complexes were analyzed by western blot for cyclin D3, cyclin D2, cyclin D1, and Fbxl8. **c** Lysates from HEK293T cells co-transfected with Flag-tagged cyclin D3 or cyclin D3Thr283A (D3TA), with HA-Fbxl8, and treated with or without MG132 (20 μM) for 4 h were subjected to immunoprecipitation with normal IgG or anti-HA. Immune complexes were analyzed by western blot for cyclin D3 and Fbxl8. **d** Schematic model of deletion mutants of Fbxl8. **e** Lysates from NIH3T3 cells co-transfected with HA tagged GFP or Fbxl8 mutants described in **d** with Flag-D3, and treated with a proteasome inhibitor MG132 (20 μM) for 4 h were precipitated with anti-Flag beads. Immune complexes were analyzed by western blot. **f** Lysates from NIH3T3 cells co-transfected with Flag-GFP or Flag-Fbxl8 with or without treatment of MG132 (20 μM) for 4 h were analyzed by western blot for cyclin D3, Fbxl8, and βactin. The numbers indicate quantifications of cyclin D3 normalized by βactin. **g** Lysates from NIH3T3 cells co-transfected with cyclin D3 or cyclin D3Thr283A (D3TA) and empty plasmid (Control) or Flag-Fbxl8, and treated with 100 μg/mL of cycloheximide (CHX) for the indicated time periods were analyzed by western blot using antibodies against cyclin D3, Fbxl8 and βactin. The numbers indicate quantifications of cyclin D3 normalized by βactin. **h** Lysates from NIH3T3 cells transfected with siControl or siFbxl8 for 3 days were treated with 100 μg/mL of cycloheximide for the indicated time periods and analyzed by western blot for cyclin D3, Cyclin D2, and Fbxl8, βactin and Cul4A. **i** Quantitative analysis of cyclin D3 from **h** to determine half-life. Data represent mean ± SD, *p < 0.05 (two-tailed Student’s *t* test, *n* = 3).

To identify the E3 ligase that regulates Thr-283 phosphorylation-dependent ubiquitylation of cyclin D3, we expressed tagged wild type cyclin D3 or cyclin D3T283A alleles in NIH3T3 fibroblasts and used mass spectrometry to identify co-purifying proteins. Fbxl8 was enriched with cyclin D3 but not cyclin D3T283A. We confirmed that Fbxl8 forms an SCF complex in vivo and in vitro (Figs. 1a and S1C) and subsequently demonstrated that endogenous Fbxl8 binds to cyclin D3 in NIH3T3 cells and Burkitt’s lymphoma, Raji cells (Figs. 1b and S1D–E). Since cyclin D2 and D3 are both expressed in lymphoid cells and exhibit analogous regulation [16], we tested whether Fbxl8 interacts with cyclin D2. Immunoprecipitation revealed that endogenous Fbxl8 co-precipitates with cyclin D3 and no specific association was apparent with cyclin D1 or D2 (Figs. 1b and S1E). It is worth noting that cyclin D2 and cyclin D3 were properly phosphorylated under these immunoprecipitation conditions (Fig. S1F). To further examine the contribution of cyclin D3 phosphorylation for binding to Fbxl8, we assessed binding of cyclin D3T283A to Fbxl8. Co-immunoprecipitation revealed weak to undetectable binding of Fbxl8 with D3T283A (Fig. 1c). To identify the domain of Fbxl8 that recognizes cyclin D3, Fbxl8 deletion mutants were generated (Fig. 1d). HA-Fbxl8 (wild type, ΔF, ΔC1, ΔC2, and ΔC3) or HA-GFP was transfected into NIH3T3 cells along with Flag-D3; reduced binding of cyclin D3 to Fbxl8ΔC3 was observed (Fig. 1e). Consistently, in vitro binding revealed direct binding of Fbxl8 to cyclin D3, but not to cyclin D2 (Fig. S1G). In addition, no binding of cyclin D3 with Fbxo4, an ubiquitin ligase for cyclin D1 [17], was observed (Fig. S1G).

### Fbxl8 regulates cyclins D3 protein stability

We next assessed whether Fbxl8 regulates cyclin D3 protein stability. Overexpression of Fbxl8 resulted in reduced expression of cyclin D3 relative to control, in an MG132-dependent manner (Fig. 1f). Fbxl8 overexpression accelerated degradation of cyclin D3 and this rapid turnover was not apparent with cyclin D3T283A (D3TA) (Fig. 1g). Cyclin D3 degradation was not accelerated by overexpression of Fbxl2, Fbxo4, Fbxo31 or Fbxo11 (Fig. S2A), demonstrating specificity. Consistently, knockdown of Fbxl8 increased basal levels of endogenous cyclin D3 (Fig. 1h). Cycloheximide (CHX) chase experiments revealed that knockdown of Fbxl8 extended half-life of cyclin D3 while cyclin D2 protein stability was unaffected (Fig. 1h, i). These results demonstrate that Fbxl8 regulates the stability of cyclin D3 in a phosphorylation-dependent manner.

### Fbxl8 catalyzes polyubiquitylation for cyclin D3

**Fig. 2:**
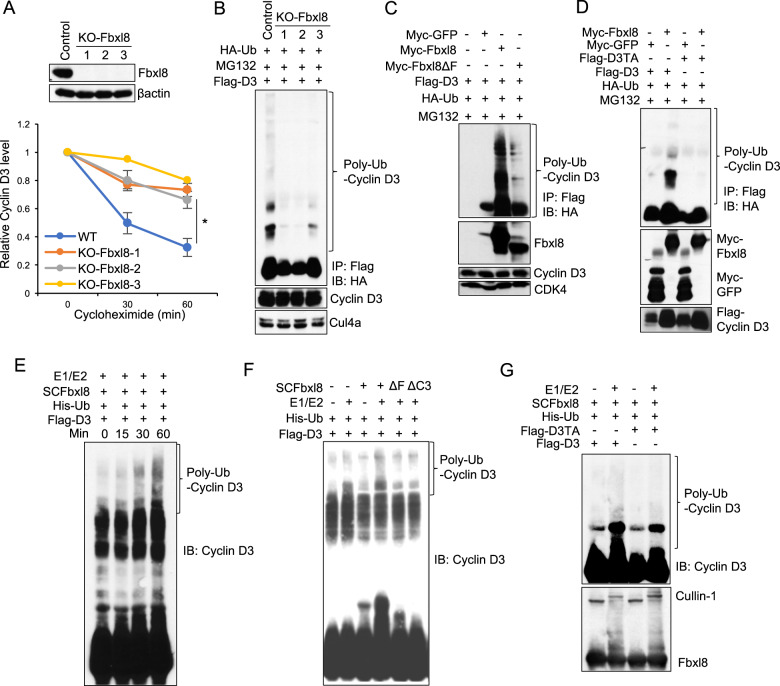
Fbxl8 ubiquitylates cyclin D3 in a phosphorylation-dependent manner. **a** Lysates from parental NIH3T3 cells versus clones (1, 2, and 3) with CRISPR/Cas9 mediated knock out of Fbxl8, were analyzed by western blot for Fbxl8 and βactin (top panel). Lysates from NIH3T3 cells or KO-Fbxl8 clones from (top panel) were treated with 100 μg/mL of cycloheximide for the indicated time periods and analyzed by western blot for cyclin D3 and βactin. Quantitative analysis of cyclin D3 is shown. Data represent mean ± SD, *p < 0.05 (two-tailed Student’s *t* test, *n* = 3). **b** Lysates from NIH3T3 (Control) or NIH3T3 KO-Fbxl8 clones were co-transfected with HA-Ub and Flag-D3 for 40 h, and treated with MG132 (20 μM) for 4 h prior to immunoprecipitation with anti-Flag beads. Immune complexes were analyzed by western blot for ubiquitinated proteins (α-HA), cyclin D3 and Cul4a as a loading control. **c**, **d** Lysates from HEK293T cells co-transfected with indicated plasmids were treated with MG132 (20 μM) for 4 h prior to immunoprecipitation with anti-Flag beads. Immune complexes were analyzed by western blot for HA-ubiquitylated proteins, total Fbxl8, cyclin D3 and CDK4. **e**–**g** In vitro ubiquitylation assays were performed in reaction mixtures containing the presence or absence of the indicated reaction mixture components. Lysates from assays were analyzed by western blot using antibodies against indicated antibodies.

To further test the function of Fbxl8, we established CRISPR-mediated knockout cell lines (clones 1, 2 and 3) in NIH3T3 cells (Fig. 2a top). Stabilization of cyclin D3 was noted in Fbxl8 KO cell lines (Fig. 2a bottom; Fig. S2B). To assess mechanisms, cells were transfected with vectors encoding Flag-D3 together with HA-Ub+MG132. No ubqiutylation of cyclin D3 was observed in KO-Fbxl8 clones, while polyubiquitin of cyclin D3 was observed in control cells (Fig. 2b). Ectopic Fbxl8 restored polyubiquitylation of cyclin D3 while Fbxl8ΔF was impaired in polyubiquitylation of cyclin D3 (Fig. 2c). Consistent with phosphorylation-dependence, wild type cyclin D3 was polyubiquitylated by Fbxl8 while the cyclin D3TA mutant was refractory (Fig. 2d). Finally, we demonstrated that recombinant SCF-Fbxl8 purified from Sf9 cells, polyubiquitylated cyclin D3, but not cyclin D2, in an F-box-dependent manner demonstrating cyclin D3 is a direct substrate (Figs. 2e–g and S2C).

### Fbxl8 loss accelerates G1-S phase transition

**Fig. 3:**
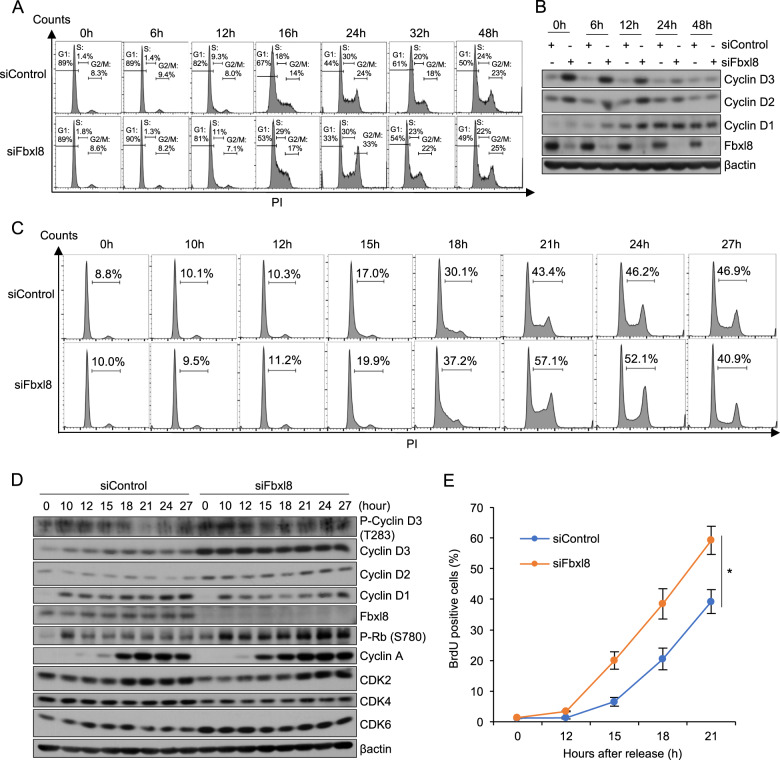
Knockdown of Fbxl8 promotes G1-S phase transition of cell cycle. **a** NIH3T3 cells were transfected with siControl or siFbxl8 and arrested at G0/G1 by contact inhibition for 36 h. Following release by replating at low density, the cell cycle was analyzed at 6, 12, 16, 24, 32, 48 h by FACS. **b** Western analysis of lysates from **a**. **c** NIH3T3 cells were transfected with either siControl or siFbxl8 and arrested at G0/G1 by contact inhibition for 36 h. Following release by replating at low density, the cell cycle was analyzed at 10, 12, 15, 18, 21, 24, and 27 h by FACS. Representative FACS profiles were shown. **d** Western analysis of lysates from **c** for phospho-cyclin D3, cyclin D3, cyclin D2, cyclin D1, Fbxl8, phospho-Rb (S780), cyclin A, CDK2, CDK4, CDK6 and βactin. **e** NIH3T3 cells were transfected with siControl or siFbxl8 and arrested at G0/G1 phase by contact inhibition for 36 h. S phase entry was assessed by BrdU incorporation (30 min) and FACS at 0, 12, 15, 18, and 21 h post release. Quantification of BrdU positive cells; mean ± SD, *p < 0.05 (two-tailed Student’s *t* test, *n* = 3).

Since overexpression of cyclin D3 accelerates G1-S phase progression [18], we reasoned that Fbxl8 loss should accelerate G1-S phase transition. Cells with siControl or siFbxl8 were arrested in G0/G1 by contact inhibition and cell cycle reentry was analyzed at 0, 6, 12, 16, 24, 32 and 48 h following replating at subconfluence (Fig. 3a). siFbxl8 was transfected into the cells before cells were synchronized resulting in cyclin D3 accumulation at the 0 h time point (Fig. 3b). Cells arrested with equal efficiency, but siFbxl8 cells transitioned from G1 to S phase with accelerated kinetics (Fig. 3a).

To focus on the impact of Fbxl8 during the G1-S phase transition, cells with siControl or siFbxl8 were synchronized in G0/G1 and cell cycle progression was analyzed at multiple time points after release. Cells transfected with siFbxl8 progressed to S phase faster than cells with siControl due to accelerated progression of G1/S phase. As a result, we observed increased S + G2/M phase populations, eventually leading to more cells entering the next cell cycle at 27 h time point (Figs. 3c and S3A). Consistent with FACS analyses, cyclin D3 levels increased earlier in siFbxl8 cells (Fig. 3d). In addition, increased phosphorylation of Rb at Ser-780, a substrate for cyclin D-CDK4/6 was observed in siFbxl8 cells relative to control cells (Fig. 3d). This is consistent with elevated kinase activity of cyclin D/CDK4/6, reflecting increased cyclin D3 and reduced degradation of phosphorylated cyclin D3. To further establish increased kinetics of S phase entry upon knockdown of Fbxl8, we ulitized BrdU incorporation. Consistently, knockdown of Fbxl8 accelerated G1-S phase transition (Figs. 3e and S3B) and increased only the S phase population in asynchronous cells (Fig. S3C). Together, these results suggest that knockdown of Fbxl8 shortens G1 phase of the cell cycle by upregulating cyclin D3.

### Enforced expression of Fbxl8 delays G1-S phase transition

**Fig. 4:**
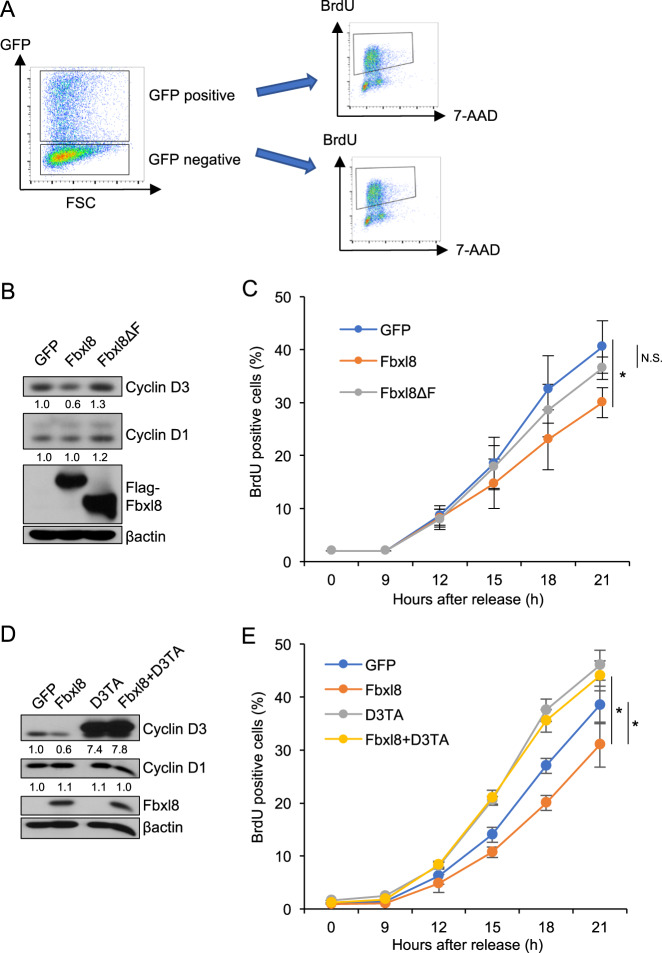
Overexpression of Fbxl8 recedes G1-S phase transition of cell cycle through degradation of cyclin D3. **a** Schematic model of experiment. NIH3T3 cells were transfected with MigR1 IRES-GFP, MigR1Fbxl8 IRES-GFP or MigR1Fbxl8ΔF IRES-GFP, and arrested at G0/G1 phase by serum starvation for 36 h. GFP positive cells and negative cells were FACS sorted and S phase entry was assessed by BrdU incorporation (30 min). **b** Western analysis of lysates from sorted GFP positive NIH3T3 cells expressing GFP, Fbxl8, or Fbxl8ΔF for cyclin D3, cyclin D1, Flag-Fbxl8 and βactin. The numbers indicate quantifications of cyclin D3 and cyclin D1 normalized by βactin. **c** GFP and BrdU double-positive cells were analyzed 9, 12, 15, 18, 21 h after release from G0/G1 phase by re-splitting cells in DMEM with 10%FBS. Quantification of GFP and BrdU double-positive cells; mean ± SD, *p < 0.05 (two-tailed Student’s *t* test, *n* = 3). N.S. Not Significant (two-tailed Student’s *t* test, *n* = 3). **d** Western analysis of lysates from sorted GFP positive NIH3T3 cells expressing GFP, Fbxl8, cyclin D3TA or Fbxl8+cyclin D3TA for cyclin D3, cyclin D1, Flag-Fbxl8 and βactin. The numbers indicate quantifications of cyclin D3, cyclin D2 and cyclin D1 normalized by βactin. **e** GFP and BrdU double-positive cells were analyzed 9, 12, 15, 18, 21 h after release from G0/G1 phase by re-splitting cells in DMEM with 10%FBS. Quantification of GFP and BrdU double-positive cells; mean ± SD, *p < 0.05 (two-tailed Student’s *t* test, *n* = 3).

Since Fbxl8 opposes cyclin D3 accumulation, we reasoned that Fbxl8 overexpression should delay cell cycle progression. A bicistronic plasmid encoding Fbxl8/IRES GFP or Fbxl8ΔF/IRES GFP was expressed in NIH3T3 cells. The bicistronic nature of this vector permits sorting on GFP positive cells that are co-expressing Fbxl8 or Fbxl8ΔF versus GFP negative cells that serve as a control for FACS assessment of cell cycle progression (Fig. 4a). We confirmed reduced expression of cyclin D3 in cells with ectopic Fbxl8, while Fbxl8ΔF had no impact on cyclin D3 (Fig. 4b). BrdU incorporation revealed that cells expressing Fbxl8 progressed to S phase slower than cells expressing only GFP, while Fbxl8ΔF mitigated Fbxl8 mediated S phase reduction (Figs. 4c and S4A). Consistently, all GFP negative populations that do not express GFP, Fbxl8 or Fbxl8ΔF progressed to S phase with similar kinetics (Fig. S4B, C). Finally, we assessed whether overexpression of Fbxl8 delays G1 phase progression through degradation of cyclin D3. Cyclin D3TA, a non-phosphorylatable mutant, was expressed together with or without Fbxl8 (Fig. 4d). BrdU incorporation revealed that cyclin D3TA hampers Fbxl8 function regulating G1-S phase (Figs. 4e and S4D). Together these results indicate that Fbxl8 regulates G1-S phase transition through degradation of cyclin D3.

### Cyclin D3 co-localizes with Fbxl8 during S-phase in the cytoplasm in a phosphorylation-dependent manner

D-type cyclins are localized in nucleus during G1 phase, where they initiate phosphorylation-dependent inactivation of Rb [12, 19, 20]; however, they translocate to the cytoplasm during S-phase as a mechanism to restrict dysregulation of cell division. We therefore assessed the subcellular localization of cyclin D3 and Fbxl8 during G1 and S phases. Cells were synchronized at G0/G1 and cell division was monitored in parallel by FACS. Immunofluorescence microscopy revealed nuclear cyclin D3 during G1 phase (8 h after release), but cytoplasmic during S phase (16 h after release). We next determined whether CRM1-dependent nuclear export of cyclin D3 is responsible for cytoplasmic localization during S-phase. Cyclin D3 re-localization to the cytoplasm was inhibited following Leptomycin B treatment consistent with CRM1-dependent nuclear export (Fig. S5A). Conversely, Fbxl8 was cytoplasmic throughout the cell cycle suggesting cyclin D3 stability during G1 phase is ensured through differential subcellular localization of cyclin D3 versus Fbxl8. Importantly, cyclin D3T283A remained nuclear during S phase consistent with p-T283 regulating cytoplasmic localization (Fig. S5B). Consistently, immunoprecipitation experiments revealed that Leptomycin B treatment prevented the binding of Fbxl8 and cyclin D3 while D3T283A is refractory to binding to Fbxl8 (Fig. S5C), indicating that p-283 coordinates nuclear export followed by its direct binding to Fbxl8 for degradation in cytoplasm.

### Cyclin D3T283A has oncogenic properties

**Fig. 5:**
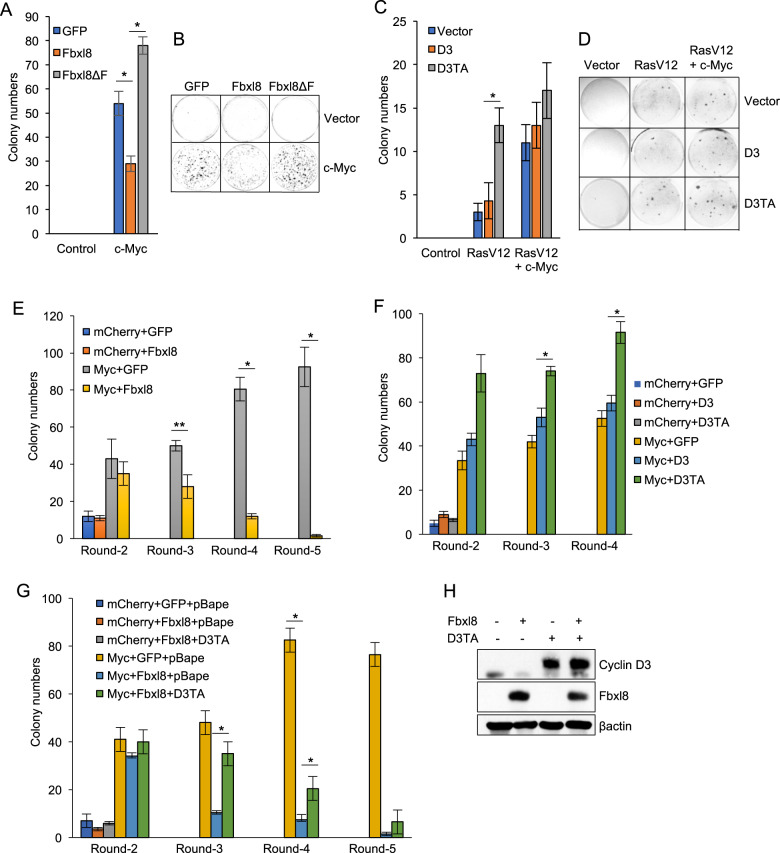
Fbxl8 suppresses c-Myc-induced transformation through phosphorylation-dependent cyclin D3 degradation. **a, b** NIH3T3 cells were co-transfected with empty vector (Vector) or c-Myc + either MigR1 (GFP), MigR1 Fbxl8 (Fbxl8) or MigR1 Fbxl8ΔF (Fbxl8ΔF), plated and cultured for 21 days. Quantification of colony numbers (**a**) and representative images (**b**). Data represent mean ± SD, *p < 0.05 (two-tailed Student’s *t* test, *n* = 3). **c**, **d** NIH3T3 cells were co-transfected with empty vector (Vector), c-Myc or c-Myc+RasV12 and MigR1 (Vector), MigR1 cyclin D3 (D3) or MigR1 cyclin D3Thr283A (D3TA), plated in soft agar medium and cultured for 21 days. Quantification of colony numbers (**c**) and representative images (**d**) are shown. Data represent mean ± SD, *p < 0.05 (two-tailed Student’s *t* test, *n* = 3). **e** Bone marrow derived HSC cells from 5-FU (150 mg/kg) treated mice were co-transduced with retrovirus encoding the indicated cDNA and plated in methycellulose media. Colony numbers were quantified and subjected to 5 rounds of serial replating. Data represent mean ± SD, *p < 0.01, **p < 0.05 (two-tailed Student’s *t* test, *n* = 3). **f** Bone marrow HSC from mice treated with 5-FU (150 mg/kg) were co-transduced with retrovirus encoding the indicated cDNA and plated in methycellulose media and colony numbers were quantified over 4 rounds of serial replating. Data represent mean ± SD, *p < 0.05 (two-tailed Student’s *t* test, *n* = 3). **g** Bone marrow HSC from mice treated with 5-FU (150 mg/kg) were co-transduced with retrovirus encoding the indicated cDNA and plated into methycellulose media and colony numbers were quantified and subjected to 5 rounds of serial replating. Data represent mean ± SD, *p < 0.05 (two-tailed Student’s *t* test, *n* = 3). **h** Lysates from **g** were analyzed by western blot using antibodies against cyclin D3, Fbxl8 and βactin.

Data thus far suggest a hypothesis wherein Fbxl8 should exhibit tumor suppressive properties reflecting its ability to antagonize cyclin D3. Because cyclin D3T283 mutations co-occur coordinately with c-myc translocation in Burkitt’s lymphoma, we assessed Fbxl8 activity in the context of c-myc overexpression. Transformation assays revealed that c-Myc-driven transformation was suppressed by Fbxl8 and enhanced by Fbxl8ΔF, suggesting tumor suppressive function of Fbxl8 (Fig. 5a, b). To address whether cyclin D3 or cyclin D3T283A has oncogenic functions and contributes to neoplastic transformation, we performed soft agar assays with D3 versus D3TA. D3TA increased H-RasV12 or H-RasV12/c-Myc-dependent transformation, while cyclin D3 was less potent (Fig. 5c, d).

Because overexpression of cyclin D3 or mutation of D3 at Thr283 is frequently observed in Burkitt’s lymphoma and leukemia [6] and c-Myc driven murine lymphomas [21, 22] (Fig. S6), we further defined whether Fbxl8 has tumor suppressive function in hematopoietic cells. Bone marrow cells isolated from 5-FU treated mice were infected with bicistronic retrovirus encoding c-Myc/Cherry, Fbxl8/GFP or c-Myc/Cherry+Fbxl8/GFP were plated on methy cellulose medium and carried through 5 rounds of replating. While cells expressing c-Myc accumulated through 5 rounds consistent with transformation, co-expression with Fbxl8 inhibited colony formation driven by c-Myc (Fig. 5e), indicating Fbxl8 is a tumor suppressor. We next assessed whether Fbxl8-dependent growth suppression is cyclin D3-dependent. Consistently, cells overexpressing c-Myc accumulated through 4 rounds, whereas co-expression with cyclin D3T283A remarkably enhanced colony formation, in contrast with cyclin D3 which was less effective (Fig. 5f). Finally we assessed whether cyclin D3T283A rescues the Fbxl8 mediated reduction in colony formation that is driven by c-Myc. Cells expressing c-Myc or cMyc+Fbxl8 formed same numbers of colonies through round 2, but by round 3–4 Fbxl8 dramatically diminished c-Myc induced colony formation (Fig. 5g). Critically, cyclin D3T283A significantly rescued Fbxl8 mediated hematopietic cell growth attenuation and this rescue was maximized at round 3 (Fig. 5g). We confirmed developed colonies still maintain the expression of cyclin D3T283A and Fbxl8 (Fig. 5h).

### Fbxl8 attenuates lymphoma cell growth and lymphoma formation in vivo

**Fig. 6:**
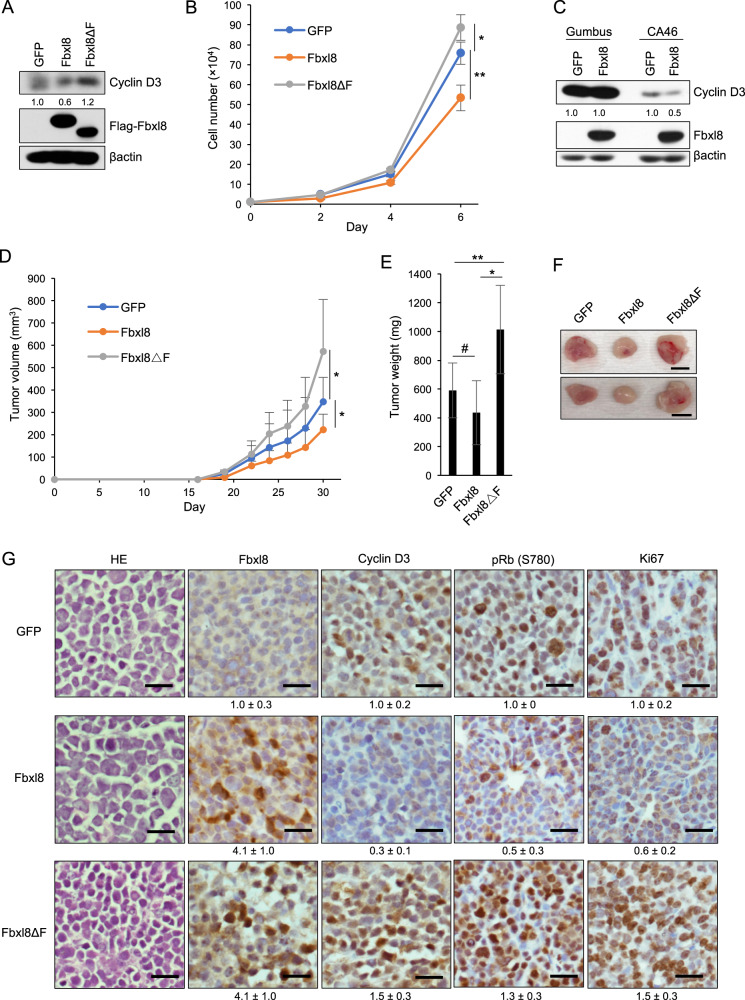
Fbxl8 suppresses lymphoma growth in vivo. **a** Lysates from CA46 cells infected with GFP, GFP/Flag-Fbxl8, or GFP/Flag-Fbxl8ΔF were analyzed by western blot for Flag-Fbxl8, cyclin D3 and βactin. The numbers indicate quantifications of cyclin D3 normalized by βactin. **b** 1 × 10^4^ CA46 cells from **a** were plated at low density and cell numbers were counted every 2 days. Data represent mean ± SD, *p = 0.02, **p < 0.01 (two-tailed Student’s *t* test, *n* = 4). **c** Lysates from Gumbus cells and CA46 cells infected with GFP, GFP/Flag-Fbxl8 were analyzed by western blot for Flag-Fbxl8, cyclin D3 and βactin. The numbers indicate quantification of cyclin D3 normalized by βactin. **d** 2 × 10^6^ CA46 cells from **a** were subcutaneously injected into 8-weeks-old SCID mice with matrigel. Tumor volumes were measured by caliper every 2 days after tumors developed, and calculated by the following formula V = (Length × width × height)/2. Data represent mean ± SD, *p = 0.02 (two-tailed Student’s *t* test, *n* = 10). **e** The average of tumor weight from **d**. Data represent mean ± SD, ^#^*p* = 0.02, **p = 0.03, *p < 0.01 (two-tailed Student’s *t* test, *n* = 10). **f** Representative images of the xenograft tumors from **e**. Scale bar indicates 1 cm. **g** Representative H&E staining and IHC staining images for Fbxl8, cyclin D3, pRb (S780), and Ki67 from **f**. Scale bar indicates 50 μm. The numbers indicate quantification of intensity determined by IHC scoring from three independent experiments. The IHC score in each experiment was defined by the following formula: Intensity = [staining positive population (1 to 3) × staining intensity (1 to 3)].

To address tumor suppressive function of Fbxl8 in an in vivo model, a Burkitt’s lymphoma cell line CA46 expressing GFP, GFP/Fbxl8, or GFP/Fbxl8ΔF was generated (Fig. 6a). CA46 were chosen as they will form xenograft tumors [21] and retain wild type cyclin D3, maintaining phosphorylation-dependent regulation of cyclin D3 by Fbxl8. Fbxl8 overexpression significantly suppressed lymphoma cell proliferation whereas Fbxl8ΔF promotes cell proliferation relative to GFP control (Fig. 6b). While Fbxl8 overexpression has no impact on cyclin D3 levels or cell proliferation in Gumbus cells (Gumbus harbor an endogenous cyclin D3 T283A mutant), Fbxl8 overexpression in CA46 downregulates wild type cyclin D3 and attenuates cell proliferation (Figs. 6c and S7A). Conversely, knockdown of Fbxl8 promoted lymphoma cell proliferation with upregulated cyclin D3 expression (Fig. S7B, C). Immune compromised mice injected with CA46 cells expressing GFP, Fbxl8, or Fbxl8ΔF were monitored every 2 days for tumor progressoin. CA46 cells with Fbxl8 overexpression formed tumors slower than cells expressing GFP and cells expressing Fbxl8ΔF developed tumors faster than cells expressing GFP, indicating tumor suppressive function of Fbxl8 and consistent with cell growth curve (Fig. 6d–f). H&E staining revealed that tumors exhibited typical histology of lymphoma (Fig. 6g). IHC staining for Fbxl8 and cyclin D3 revealed that tumors maintained Fbxl8 or Fbxl8ΔF accompanied with reduced or increased expression of cyclin D3, respectively (Fig. 6g). While Fbxl8 overexpression resulted in reduced phospho-Rb (S780) and Ki67 in xenograft tumors, Fbxl8ΔF increased positivity of phospho-Rb (S780) and Ki67 (Fig. 6g). We finally tested whether Fbxl8 reduces lymphoma proliferation through degradation of cyclin D3. Cyclin D3TA was expressed together with or without Fbxl8 (Fig. S7D). Cyclin D3TA overcomes Fbxl8 mediated attenuation of lymphoma proliferation (Fig. S7E). Together these results demonstrate that Fbxl8 functions as a tumor suppressor through degradation of cyclin D3 in lymphoma.

### Fbxl8 is negatively correlated with cyclin D3 in human lymphomas

**Fig. 7:**
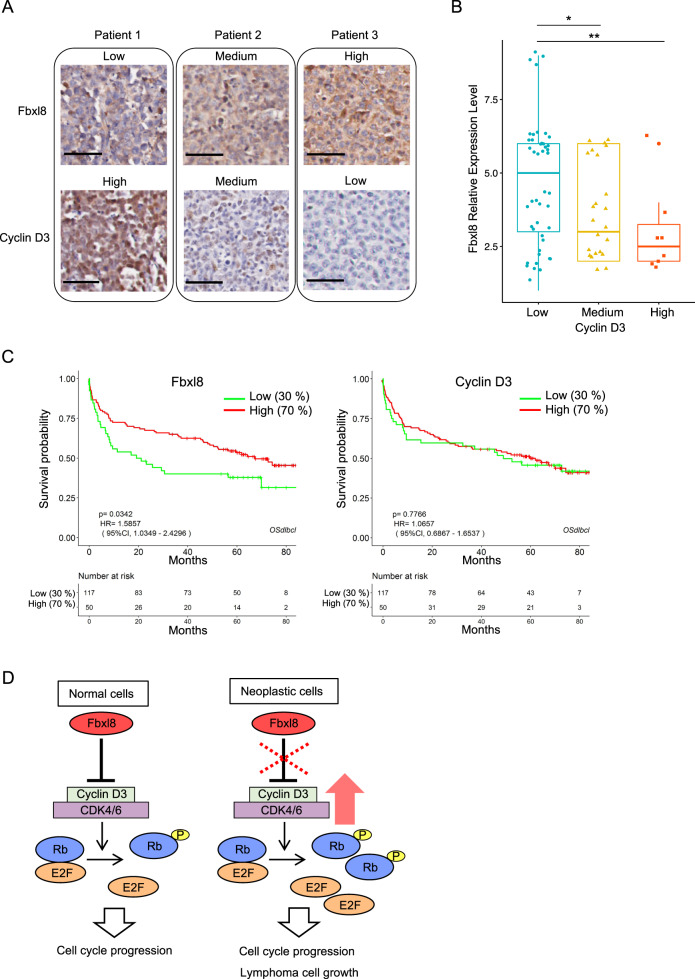
Fbxl8 expression is negatively correlated with cyclin D3 expression in human lymphomas. **a** Representative IHC staining images from human patients for low, medium, and high expression of cyclin D3 or Fbxl8. Scale bar indicates 50 μm. **b** Each IHC score was calculated as described in the Materials & Methods. The box plot with scatters and statistics are shown, *p = 0.046, **p = 0.017 (Wilcoxon rank sum test, *n* = 77). **c** Kaplan–Meier survival curve for overall survival in lymphoma patients with low and high expression of Fbxl8 (left panel) or cyclin D3 (right panel). **d** Model of lymphoma cell growth by Fbxl8-cyclin D3 axis.

We subsequently evaluated the relevance of the Fbxl8-cyclin D3 axis in human patients. Normal lymph node tissues, spleen, tonsil, and lymphomas arrayed on slides (US Biomax, Inc.) were stained with cyclin D3 and Fbxl8 specific antibodies (Fig. 7a) and IHC scores of each core were calculated (details in Materials & Methods). We categorized cyclin D3 expression as low, medium, and high in all samples and plotted on the x axis to compare with IHC scores for Fbxl8 (Fig. 7a, b). Patient samples with low, medium, or high expression of cyclin D3 have high, medium, or low expression of Fbxl8, respectively, underscoring Fbxl8 negatively regulates cyclin D3 expression in human lymphomas in a dose dependent manner. We further used a recently developed online tool to assess the lymphoma patients’ overall survival with gene expression [23], and observed that the low expression of Fbxl8 mRNA significantly correlated with reduced overall survival for lymphoma patients while the expression of cyclin D3 mRNA does not (Fig. 7c). We suggest this is consistent with our conclusions that Fbxl8 regulates cyclin D3 at posttranslational level and Fbxl8 has a tumor suppressive function. This further suggested that cyclin D3-dependent kinase might be a novel therapeutic target in certain lymphomas that harbor low expression of Fbxl8 or high expression of cyclin D3.

### Cyclin D3 overexpression renders cells to susceptible to CDK4/6 small molecule inhibitors

CDK4/6 inhibitors (CDK4/6i) have been evaluated in clinical trials in many cancer types with promising results [24]. Given that cyclin D3TA has oncogenic properties and promotes oncogenic-driven transformation (Fig. 5), we assessed the efficacy of the CDK4/6i, palbociclib, on cells expressing wild type versus mutant cyclin D3. We confirmed robust expression of both cyclin D3 and cyclin D3TA (Fig. S8A). As expected cyclin D3TA expression is elevated relative to wild type cyclin D3, reflecting increased stability of cyclin D3TA by circumventing phosphorylation-dependent degradation by Fbxl8 while mRNA level is unchanged between wild type cyclin D3 and cyclin D3TA (Fig. S8A, B). While NIH3T3 cells expressing vector control were relatively refractory to palbociclib, those overexpressing either cyclin D3 or cyclin D3TA were sensitive to palbociclib and arrested at G1 (Fig. S8C, D), consistent with a previous report that palbociclib-induced senescence is linked to CDK4 hyperactivation [25].

### GSK3β does not phosphorylate cyclin D3 for degradation

Previous reports implicated p38 as a potential kinase that phosphorylates cyclin D3 at Thr-283 [26]. We assessed whether p38 affects cyclin D3 expression in CA46 Burkitt’s lymphoma cells. Surprisingly, SB203580 (p38 inhibitor) did not significantly impact cyclin D3 (Fig. S9A). The GSK3β inhibitor SB216763 marginally increased cyclin D3 protein level and slightly reduced p-cyclin D3 (T283) (Fig. S9A). Given that D-type cyclins function during G1-S phase transition of the cell cycle, we next assessed the impact of p38 on cyclin D3 expression during G1-S phase. We confirmed that cells either with or without treatment of inhibitors entered S phase 15 h after release from G1 phase (Fig. S9B). While SB216763 (GSK3β inhibitor) increased cyclin D1 expression in late G1 to S phase as we previously demonstrated [20], SB203580 and SB216763 had a marginal impact on cyclin D3 (Fig. S9C). The collective data suggest that the kinase that regulates cyclin D3 remains to be identified conclusively.

## Discussion

D-type cyclins function as mitogenic sensors that integrate growth factor signaling with cell division and proliferation. It is therefore of little surprise that all three D-type cyclins are dysregulated in human cancers. Cyclin D3 is overexpressed in specific human cancers and is subject to point mutations specifically in Burkitt’s lymphoma [6]; the specific residue targeted, Thr-283 has been implicated in regulating cyclin D3 protein destruction [26]. Given the prevalence of D3 mutations in Burkitt’s lymphoma, it is striking that little is known about post-translational modification of cyclin D3. We have therefore identified Fbxl8 as the specificity subunit of an SCF E3 ligase that specifically recognizes and polyubiquitylates Thr-283 phosphorylated cyclin D3 thereby triggering its proteasome-dependent degradation (Fig. 7d). It is of importance to note that while SCF-Fbxl8 neither binds nor regulates accumulation of cyclin D1, our data support a role for SCF-Fbxl8 in regulating cyclin D2 indirectly. We were unable to observe the direct interaction of cyclin D2 and Fbxl8, and to reconstitute direct ubiquitylation of cyclin D2 with SCF-Fbxl8 suggesting that while it can regulate cyclin D2 in vivo indirectly, there is either another ubiquitin-ligase that specifically regulates its degradation or a critical secondary specificity factor that specifies cyclin D2 is yet to be identified.

Functional analyses using either knockdown of Fbxl8 or overexpression of wild type or mutant Fbxl8 demonstrated its role in regulating the G1-S phase transition. This result is consistent with its role as a negative regulator of cyclin D3 given that overexpression of either cyclin D accelerates the G1-S phase transition [18]. Importantly, cyclin D3T283A, a non-phosphorylable mutant, rescued Fbxl8 overexpression mediated cell proliferation attenuation, indicating that Fbxl8-cyclin D3 axis plays a critical role in regulating lymphoma cell proliferation. However, cyclin D3T283A mutant partially rescued Fbxl8 induced hematopietic cell growth inhibition (Fig. 5g). This suggests that Fbxl8 may have other substrates that promote hematopietic transformation. While SCF-Fbxl2 has been implicated as a potential regulator of cyclin D3, regulation by Fbxl2 is independent of cyclin phosphorylation [27, 28]. More importantly, inactivation of Fbxl2 results in a mitotic defect suggesting its predominant substrate functions during mitosis, independent of either cyclin D3 or D2 although we observed the binding of Fbxl2 and cyclin D3 (Fig. S1D).

Fbxl8 overexpression dramatically suppressed oncogene-induced transformation in vitro and lymphoma progression in vivo. Consistently, we found that cyclin D3 expression is negatively correlated with Fbxl8 expression in human lymphomas and low level of Fbxl8 correlates poor overall survival for lymphoma patients, supporting our conclusion that Fbxl8-cyclin D3 axis has a critical role in regulating cell cycle and tumorigenesis.

CDK4/6 inhibitors are being used for many clinical trials and the efficacy has been assessed in many types of cancers where cyclin D1 is overexpressed [24, 29–31]. Our data demonstrate that cyclin D3T283A accumulates in the nucleus and promotes oncogene-induced tumorigenesis in hematopoietic cells, reflecting increased CDK activity. Indeed, Rb is highly phosphorylated by knockdown of Fbxl8. Importantly, cells harboring either a D3T283A allele or overexpressing wt D3 were more responsive to palbociclib treatment than parental cells. This supports the notion that Fbxl8-cyclin D3 axis might be a potential novel biomarker to predict the efficacy of CDK4/6i in lymphomas.

In summary, Fbxl8 regulates cell cycle progression and lymphoma cell proliferation through cyclin D3. Thus, either loss of Fbxl8 or gain of a cyclin D3 mutation that stabilizes cyclin D3 protein level will accelerate cell cycle progression and lymphoma cell growth consistent Fbxl8 functioning as a tumor suppressor (Fig. 7d).

## Materials and methods

### Cell culture, transfection, infection, cell cycle analyses and CRISPR-Cas9 knockout

NIH3T3 and 293T cells were cultured in Dulbecco’s modified Eagle’s medium (DMEM) supplemented with 10% fetal bovine serum (FBS), 100 units/ml of penicillin, and 100 mg/ml of streptomycin (Corning). U2OS cells were cultured in McCoy’s 5A medium supplemented with 10% fetal bovine serum (FBS), 100 units/ml of penicillin, and 100 mg/ml of streptomycin (Corning). For transfection, expression plasmids were transfected into cells with lipofectamine (ThermoFisher Scientific) or PolyJet (SignaGen Laboratories) reagents according to the manufacturer’s instructions. For siRNA transfection, siRNA was introduced into cells with Lipofectamine RNAiMAX (ThermoFisher Scientific) according to the manufacturer’s instructions. siRNA for control and Fbxl8 (SMARTpool: siGENOME Fbxl8 siRNA) were purchased from Dharmacon. For viral production, viral expression plasmids and packaging plasmids were co-transfected into 293T cells with lipofectamine (ThermoFisher Scientific). Virus supernatants harvested 48–72 h after transfection were used to infect cells with polybrene (10 μg/mL). See supplementary material for cell cycle analyses and CRISPR-Cas9 knockout.

### Western analyses and Mass spectrometry analysis

Cells were lysed in EBC buffer (50 mM Tris pH 8.0, 120 mM NaCl, 1 mM EDTA, 0.5% NP40) containing 1 mM phenylmethylsulfonyl fluoride, 20 U/ml aprotinin, 1 μM leupeptin, 1 mM dithiothreitol, 0.1 mM NaF, 0.1 mM Sodium orthovanadate, and 10 mM β-glycerophosphate. Proteins were resolved by SDS-PAGE, transferred to membrane, and immunoblotted with the indicated antibodies. See supplementary material for information of antibodies. Membranes were incubated with horseradish peroxidase-conjugated anti-mouse or rabbit antibodies and signals were developed with the ECL system (PerkinElmer) according to the manufacturer’s instructions. See supplementary material for mass spectrometry analysis.

### In vitro binding assay

1 × 10^6^ sf9 insect cells were infected with baculovirus expressing Flag-Fbxl8 or Flag-Fbxo4 for 1 h and harvested 40 h post infection. 1 × 10^6^ sf9 insect cells were infected with baculovirus expressing cyclins along with CDK4 for 1 h and harvested 40 h after infection. Harvested cells were lysed in Tween20 lysis buffer (50 mM HEPES (pH 8.0), 150 mM NaCl, 2.5 mM EGTA, 1 mM EDTA, 0.1% Tween 20) with inhibitors for protease and phosphatase (1 mM PMSF, 20 U/ml aprotinin, 5 mg/ml leupeptin, 1 mM DTT, 0.4 mM NaF, 10 mM b-glycerophosphate, and 100 nM okadaic acid) for 30 min. Flag-Fbxl8 or Flag-Fbxo4 was immunoprecipitated from sf9 lysates using anti Flag beads (Sigma Cat No. A2220). The beads were washed with Tween20 lysis buffer and Flag-Fbxl8 or Flag-Fbxo4 was purified. Purified Flag-Fbxl8 or Flag-Fbxo4 was incubated with Cyclin D3/CDK4 or cyclin D2/CDK4 purified from sf9 cells for 4 h and Flag beads were washed with Tween20 lysis buffer for 5 times. Immune complexes were analyzed by western blot.

### Ubiquitylation assay

For in vivo ubiquitination assay, NIH3T3 or HEK293T cells were transfected with indicated plasmids (Detail plasmids are described in the figure legends) for 40 h and treated with MG132 (20 μm) for 4 h. The supernatants of cell lysates in 0.2% SDS with RIPA buffer were subjected to immunoprecipitation with antibodies against HA or Flag followed by western analysis to detect the ubiquitinated proteins. For in vitro ubiquitination, SCF components including Fbxl8, Cul1, Skp1, Roc1 were purified from 293T cells and incubated with purified cyclin D3/CDK4 from sf9 cells in the presence of E1/E2 enzymes, ubiquitin, and ATP at 37 °C for 60 min.

### qRT-PCR analysis

Total RNA was isolated using the RNeasy Micro Kit (Qiagen) and reverse transcribed using iScript cDNA Synthesis Kit (Bio-Rad) according to the manufacturer’s instructions. qRT-PCR was performed with SsoAdvanced Universal SYBR Green Supermix (Bio-Rad) and the data were normalized by GAPDH.

### Immunofluorescence staining

Cells were fixed in 4% paraformaldehyde, permeabilized in 0.5% Triton X-100, stained with rabbit polyclonal and mouse monoclonal antibodies, incubated with Alexa Fluor 488 or 594-conjugated anti-mouse/rabbit IgG (Life Technologies), and mounted with ProLong Gold sntifade reagent with DAPI (Invitrogen).

### Transformation assay in soft agar

2500 cells infected with H-rasV12 or c-Myc were seeded in agarose medium (Lonza) (0.4% low melting point agarose as a lower layer and 0.8% agarose as a top layer) on 6-well plates. Cells were grown in 37 °C, 5% CO_2_ for 21–26 days and colonies were quantified.

### Colony formation assay in methylcellulose

Bone marrow cells were aseptically isolated from 5-fluorouracil (5-FU; 150 mg/kg) treated C57BL/6 mice. After red blood cells were removed by using ACK lysis buffer (Lonza), 2 × 10^4^ nucleated cells were plated in triplicates into methylcellulose medium (MethoCult3234; Stem Cell Technologies) supplemented with 50 ng/mL FLT3L, 50 ng/mL SCF, 10 ng/mL IL3, 10 ng/mL IL6, and 10 ng/mL IL7 (Stem Cell Technologies). The colony numbers were counted every 7 days and re-plated for next round of serial replating.

### Xenograft

2 × 10^6^ CA46 cells were subcutaneously injected into 8-weeks-old SCID mice with matrigel (BD). Tumor volumes were measured by caliper every 2 days and calculated by the following formula V = (length × width × height)/2. Mice were sacrificed and the tumor size was measured when tumors reached 10 mm. Care of experimental animals was in accordance with institutional guidelines.

### Immunohistochemistry staining and tissue micro array

Lymphoma tissues from xenograft were fixed with 4%PFA, dehydrated, and embedded in paraffin followed by sectioned with a microtome. H&E sections and tissue microarray slides (US Biomax, Inc.) were blocked with 10% goat serum, incubated with the primary antibodies and subsequently incubated with biotinylated antibodies. Signal was developed with ABC substrate kit (Vector) followed by DAB reaction (Vector), and counterstained with Hematoxylin (Thermo Scientific). The Ki-67 antibody (ab15580) was purchased from Abcam. Fbxl8 antibody (NBP2-34012) was obtained from Novus Biologicals. Cyclin D3 (DCS22) and pRb (S780) were purchased from Cell Signaling Technology and Santa Cruz, respectively. The IHC score in each core was defined by following formula Intensity = (staining positive population (1 to 3) × staining intensity (1 to3)). The IHC scores 1–2, 3–5, and 6–9 were defined as expression of low, medium, and high, respectively.

### Statistics, sample size and randomization

The error bars represent standard deviation (SD) of the mean and significance was defined as a *p* value less than 0.05 with two-tailed, unless otherwise specified. The normality and equal variance of data were evaluated by Shapiro–Wilk test and *F*-test, respectively. When two groups were normally distributed, the parametric test was applied such as Student’s *t* test for equal variance and Welch’s *t*-test for unequal variance. When group was not normally distributed, non-parametric test such as Wilcoxon rank-sum test was applied. At least three independent experiments were performed to obtain reproducibility with statistical significance. Sample size and *P* value were described in figure legends. All samples and animals were randomly allocated to the groups and all investigators were blinded to the group allocation during the experiments.

## Supplementary information

Supplemental methods and figures
