# Supplementary material for: Fbxl8 suppresses lymphoma growth and hematopoietic transformation through degradation of cyclin D3
Source: Oncogene. 2020 Oct 29;40(2):292–306. doi: 10.1038/s41388-020-01532-4 (PMC7808939; doi:10.1038/s41388-020-01532-4)
Supplement: Supplementary file 1 — Supplemental methods and figures [file 41388_2020_1532_MOESM1_ESM.pdf]

Methods

Cell Cycle analyses

NIH3T3 cells were synchronized in G0/G1 phase by contact inhibition or DMEM containing 0.2% FBS for 36 hours at high density. Cell cycle were released from G0/G1 phase by splitting cells with DMEM containing 10% FBS. Cells were harvested, fixed with 70% ethanol, and stained with 10μg/ml propidium iodide (PI) containing 100 μg/ml RNase A and 0.5% FBS. BrdU assay was performed according to the manufacturer's instructions (BD Biosciences). Cell cycle distribution and BrdU positive cells were measured with BD LSRFortessa (BD Biosciences).

CRISPR-Cas9 knockout

For establishment of CRISPR-Cas9 knockout cell lines, sgRNAs against Fbxl8 were cloned into lentiCRISPR v2 plasmid obtained from Addgene (#52961). The sgRNA sequences are as follows: 5'-CACCGATGAGCGCCAACACTTCCTC-3' and 5'-AAACGAGGAAGTGTTGGCGCTCATC-3'. Cells were transfected with lentiCRISPR v2 containing sgRNA against Fbxl8 and plated at low density following selection by puromycin. Knockout clones were determined by western blotting after single clone isolation.

Antibodies for Western analyses

cyclin D1 (72-13G), cyclin D2 (M20), P-Rb (S780), CDK2 (M2), CDK4 (H-303), CDK6 (C-21), PRMT5 and Fbxl8 were obtained from Santa Cruz. Cyclin D2 (D52F9), cyclin D3 (DCS22), Phospho-cyclin D3 (Thr283) (E1V6W), Skp1 (D3J4N), Cul1, Rbx1, HA

(C29F4), Myc-Tag (9B11) and GAPDH (14C10) were purchased from Cell Signaling Technology. Cul4a was purchased from Bethyl labolatories. Cyclin A, Flag (F7425) and βactin (AC-15) were purchased from Sigma. Phospho-cyclin D2 (T280) was purchased from Assay Biotech.

Mass spectrometry analysis

NIH 3T3 stably expressing Flag-tagged D3 (wild-type) or Flag-tagged D3 T283A (mutant) cells were harvested and subsequently lysed in Tween20 lysis buffer (50 mM HEPES (pH 8.0), 150 mM NaCl, 2.5 mM EGTA, 1 mM EDTA, 0.1% Tween 20) with protease and phosphatase inhibitors (1 mM PMSF, 20 U/ml aprotinin, 5 mg/ml leupeptin, 1 mM DTT, 0.4 mM NaF, 10 mM b-glycerophosphate, and 100 nM okadaic acid). Cyclin D3 complexes were purified using M2 agarose (Sigma), separated by SDS-PAGE, and stained with Coomassie Brilliant Blue. All separated bands were excised into approximately 1 mm^3 pieces. Gel pieces were then subjected to a modified in-gel trypsin digestion procedure [3]. Gel pieces were washed and dehydrated with acetonitrile for 10 min followed by removal of acetonitrile. Pieces were then completely dried in a speed-vac. Rehydration of the gel pieces was with 50 mM ammonium bicarbonate solution containing 12.5 ng/µl modified sequencing-grade trypsin (Promega, Madison, WI) at 4ºC. After 45 min, the excess trypsin solution was removed and replaced with 50 mM ammonium bicarbonate solution to just cover the gel pieces. Samples were then placed in a 37ºC room overnight. Peptides were later extracted by removing the ammonium bicarbonate solution, followed by one wash with a solution containing 50% acetonitrile and 1% formic acid. The extracts were then dried in a speed-vac (~1 hr). The samples were then stored at 4ºC until analysis. For

analysis, the samples were reconstituted in 5 - 10 µl of HPLC solvent A (2.5% acetonitrile, 0.1% formic acid). A nano-scale reverse-phase HPLC capillary column was created by packing 5 µm C18 spherical silica beads into a fused silica capillary (125 µm inner diameter x ~20 cm length) with a flame-drawn tip [2]. After equilibrating the column each sample was loaded via a Famos auto sampler (LC Packings, San Francisco CA) onto the column. A gradient was formed, and peptides were eluted with increasing concentrations of solvent B (97.5% acetonitrile, 0.1% formic acid). As peptides eluted, they were subjected to electrospray ionization and then entered into an LTQ Velos ion-trap mass spectrometer (ThermoFisher, San Jose, CA). Peptides were detected, isolated, and fragmented to produce a tandem mass spectrum of specific fragment ions for each peptide. Peptide sequences (and hence protein identity) were determined by matching protein databases with the acquired fragmentation pattern by the software program, Sequest (ThermoFisher, San Jose, CA) [1]. Spectral matches were manually examined and multiple identified peptides per protein were required.

1 Eng JK, McCormack AL, Yates JR. An approach to correlate tandem mass spectral data of peptides with amino acid sequences in a protein database. Journal of the American Society for Mass Spectrometry 1994; 5: 976-989.

2 Peng J, Gygi SP. Proteomics: the move to mixtures. Journal of mass spectrometry : JMS 2001; 36: 1083-1091.

3 Shevchenko A, Wilm M, Vorm O, Mann M. Mass spectrometric sequencing of proteins silver-stained polyacrylamide gels. Analytical chemistry 1996; 68: 850-858.

Yoshida_Fig. S1

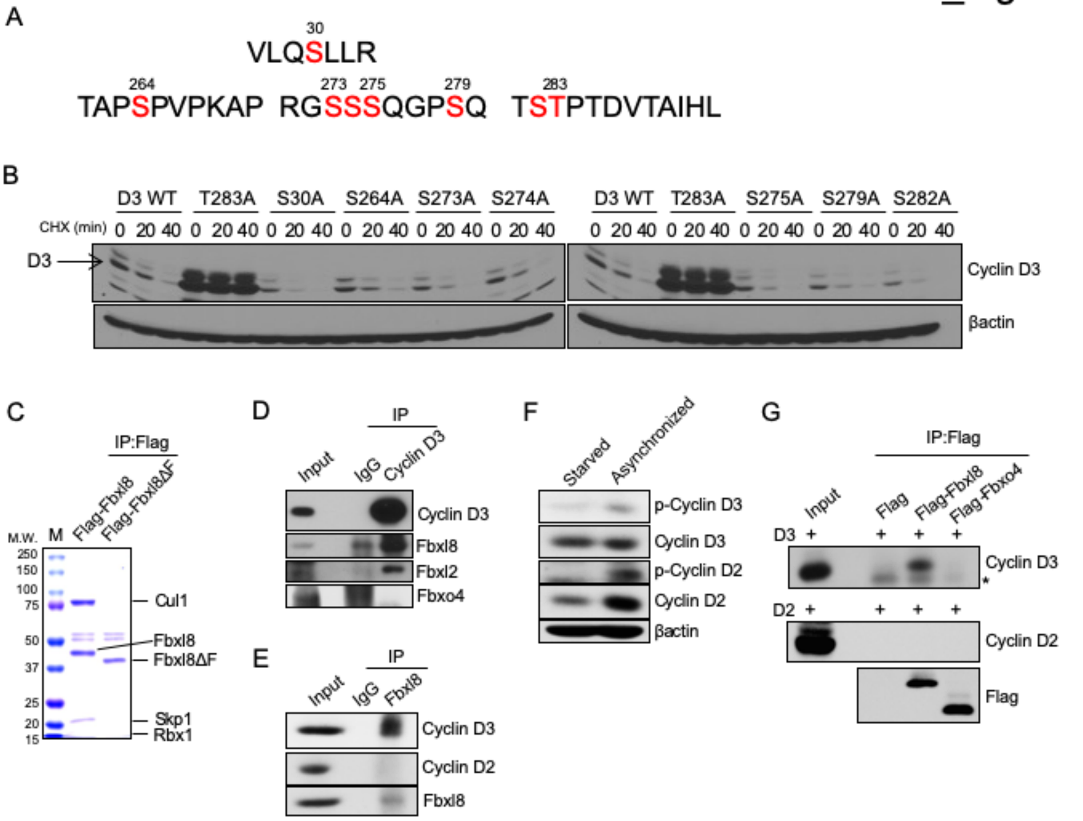

Figure S1. Fbxl8 interacts with and regulates cyclin D3 in a phosphorylation dependent manner.

(A) Phosphorylated residues (red) in cyclin D3 identified by mass spec. (B) Lysates from NIH3T3 cells transfected with cyclin D3 (WT), T283A, S30A, S264A, S273A, S274A, S275A, S279A or S282A and treated with 100 µg/mL of cycloheximide (CHX) for the indicated time periods were analyzed by western blot using antibodies against cyclin D3 and βactin. Only Thr-283 is required for rapid degradation of cyclin D3. (C) Lysates from sf9 cells infected with Cul1, Skp1, Rbx1 and Flag-Fbxl8 or Flag-Fbxl8ΔF were immunoprecipitated with anti Flag beads. Immune complexes were analyzed by Coomassie Brilliant Blue staining for SCF components (Cul1, Skp1, and Rbx1). (D) Lysates from NIH3T3 cells treated with a proteasome inhibitor MG132 (20 µM) for 4 hours were immunoprecipitated with antibodies against normal IgG or cyclin D3. Immune complexes were analyzed by western blot for cyclin D3, Fbxl8, Fbxl2 and Fbxo4. (E) Lysates from Raji cells treated with a proteasome inhibitor MG132 (20 µM) for 4 hours were immunoprecipitated with antibodies against normal IgG or Fbxl8. Immune complexes were analyzed by western blot for cyclin D3, cyclin D2 and Fbxl8. (F) Lysates from serum starved and asynchronized NIH3T3 cells were analyzed by western blot using antibodies against p-cyclin D3 (T283), cyclin D3, p-cyclin D2 (T280), cyclin D2 and βactin. (G) Immune complexes purified from sf9 cells infected with empty vector (Flag), Flag-Fbxl8 or Flag-Fbxo4 by immunoprecipitation with anti Flag beads were incubated with lysates from from sf9 cells infected with cyclin D3/CDK4 or cyclin D2/CDK4 for 4 hours. *In vitro* binding was assessed by western blot for cyclin D3, cyclin D2 and Flag. * indicates a nonspecific band.

Yoshida_Fig. S2

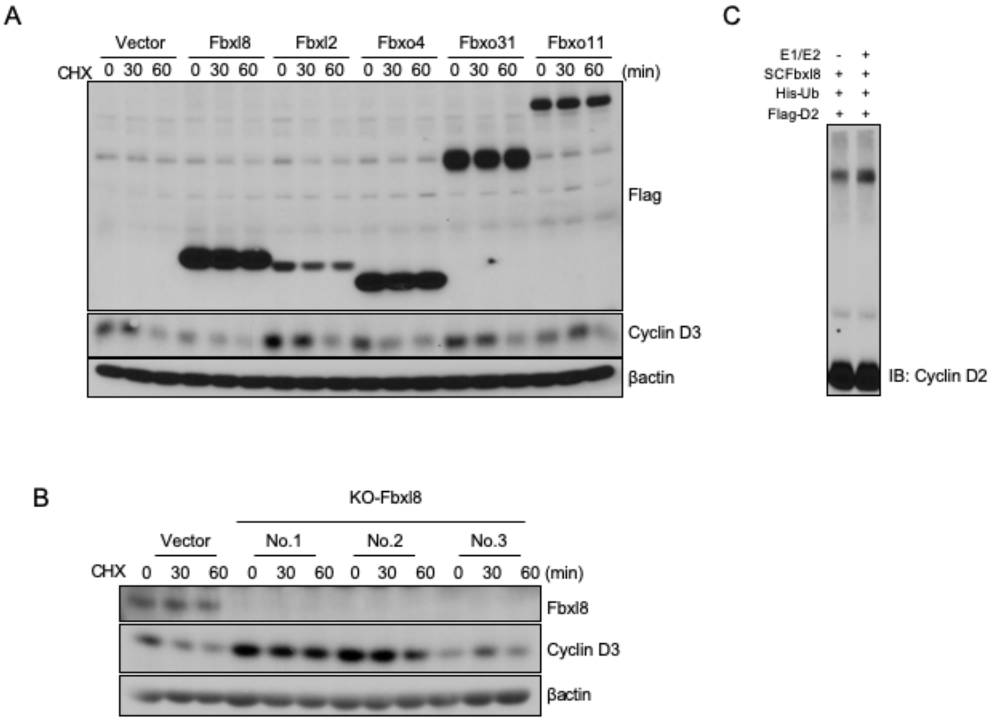

Figure S2. Fbxl8 regulates cyclin D3 stability.

(A) Lysates from U2OS cells transfected with ectopic Fbxl8, Fbxl2, Fbxo4, Fbxo31, or Fbxo11 and treated with cycloheximide (CHX) as indicated time periods were analyzed by western blot using antibodies against Flag, cyclin D3, cyclin D2, cyclin D1, and βactin. (B) Lysates from CRISPR-mediated knockout cell lines (clones 1, 2 and 3) in NIH3T3 cells treated with CHX as indicated time periods were analyzed by western blot using antibodies against Fbxl8, cyclin D3, cyclin D2, cyclin D1, and βactin. (C) *In vitro* ubiquitination assays were performed in reaction mixtures containing the presence or absence of the indicated reaction mixture components. Lysates from assays were analyzed by western blot using an antibody against cyclin D2.

Yoshida_Fig. S3

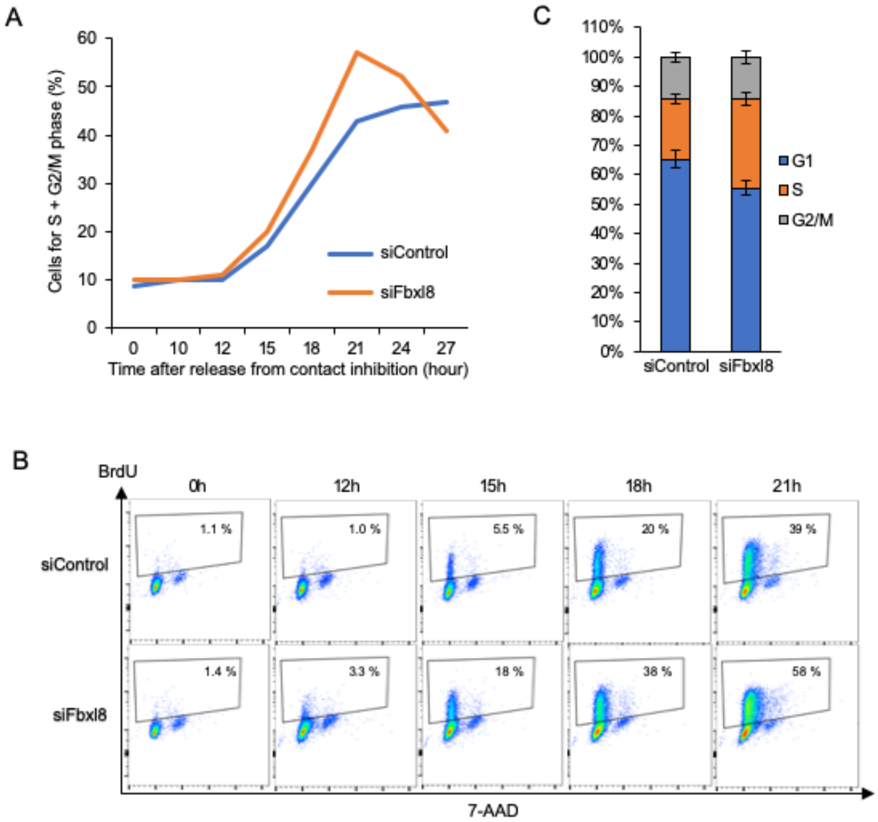

Figure S3. Fbxl8 regulates the G1-S phase transition.

(A, B) NIH3T3 cells were transfected with siControl or siFbxl8 and arrested at G0/G1 by contact inhibition for 36 hours. (A) Cell cycle was analyzed 10, 12, 15, 18, 21, 24, and 27 hours after release from G0/G1 phase. Quantification of cells in S+G2/M phase was shown. (B) S phase entry was assessed by BrdU incorporation assay (30 min) using cells 0, 12, 15, 18, and 21 hours after release from G0/G1 phase. Representative FACS profiles were shown. (C) NIH3T3 cells were transfected with siControl or siFbxl8. Cell cycle distribution was measured by BrdU incorporation assay.

Yoshida_Fig. S4

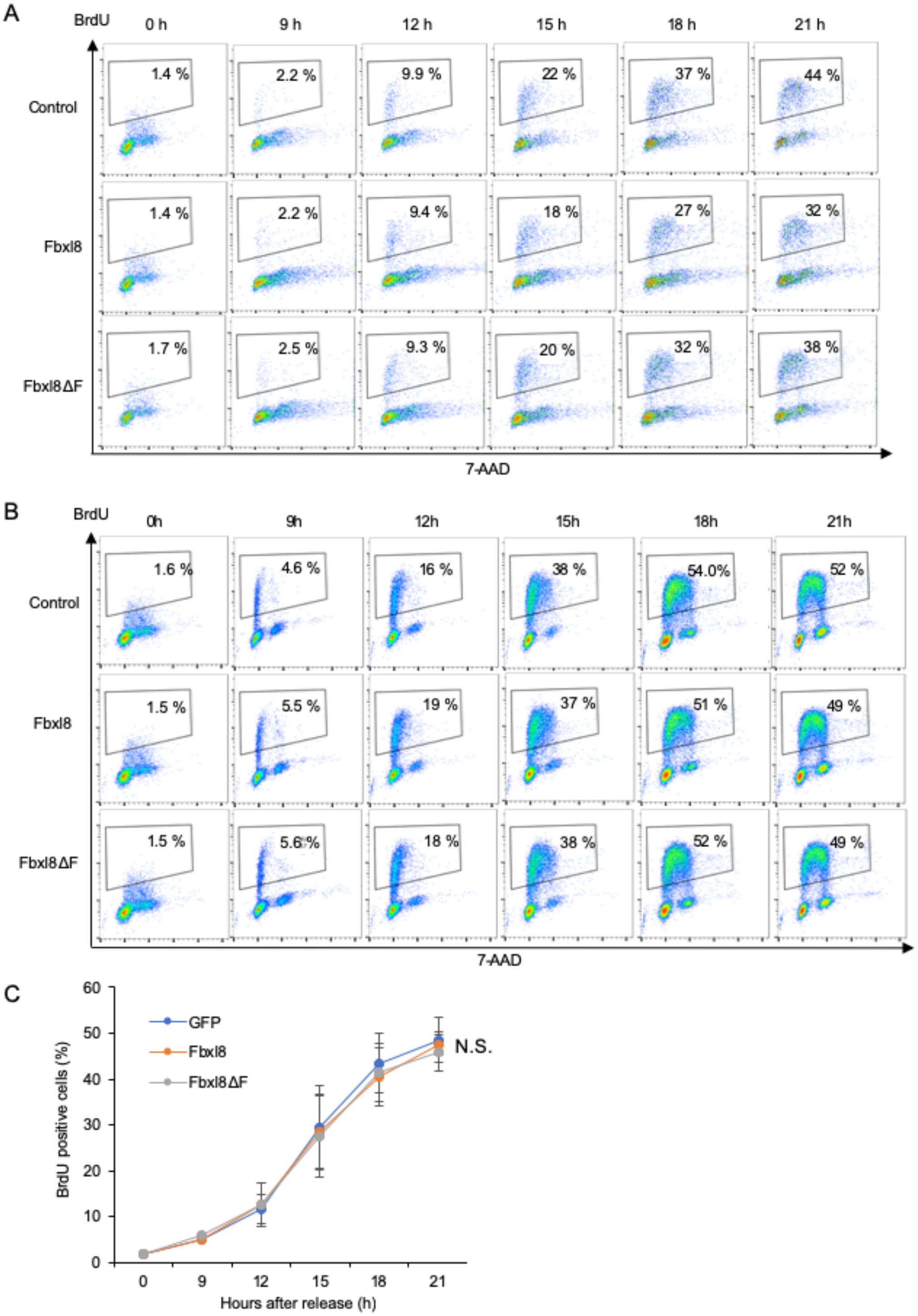

D

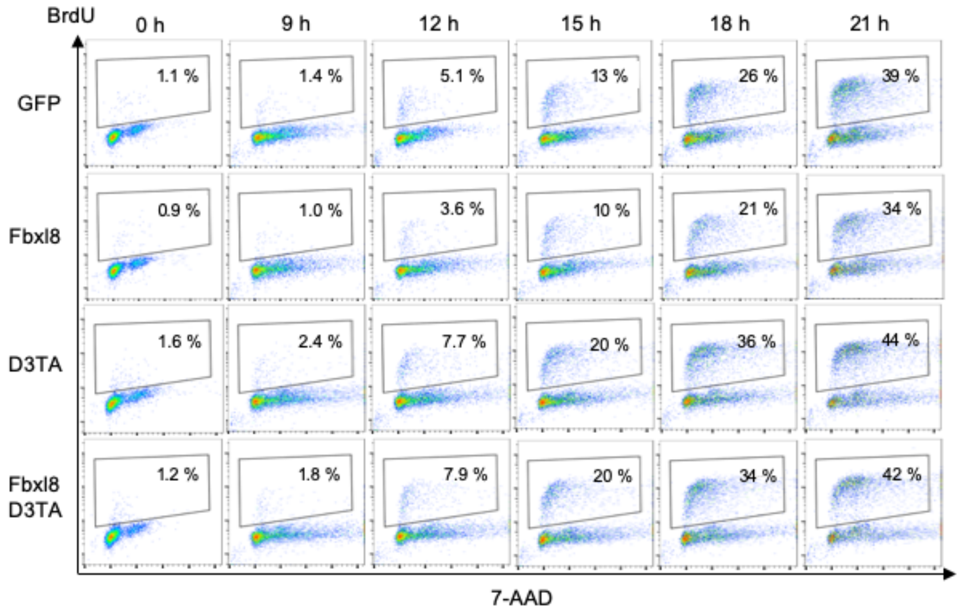

Figure S4. Fbxl8 regulates the G1-S phase transition through cyclin D3.

(A, B) NIH3T3 cells were transfected with MigR1 IRES-GFP, MigR1Fbxl8 IRES-GFP or MigR1Fbxl8ΔF IRES-GFP, and arrested at G0/G1 phase by serum starvation for 36 hours. GFP and BrdU double-positive cells (A) and GFP negative and BrdU positive cells (B) were analyzed 0, 9, 12, 15, 18, 21 hours after release from G0/G1 phase by re-splitting cells in DMEM with 10%FBS. Representative FACS profiles were shown. (C) Quantification of GFP negative and BrdU positive cells from (B); mean ± SD, N.S., Not Significant (two-tailed Student's t-test, n=3). (D) NIH3T3 cells were transfected with MigR1 IRES-GFP, MigR1Fbxl8 IRES-GFP, MigR1cyclin D3TA IRES-GFP, or MigR1Fbxl8 IRES-GFP + MigR1cyclin D3TA IRES-GFP and arrested at G0/G1 phase by serum starvation for 36 hours. GFP and BrdU double-positive cells were analyzed 9, 12, 15, 18, 21 hours after release from G0/G1 phase by re-splitting cells in DMEM with 10%FBS. Representative FACS profiles were shown.

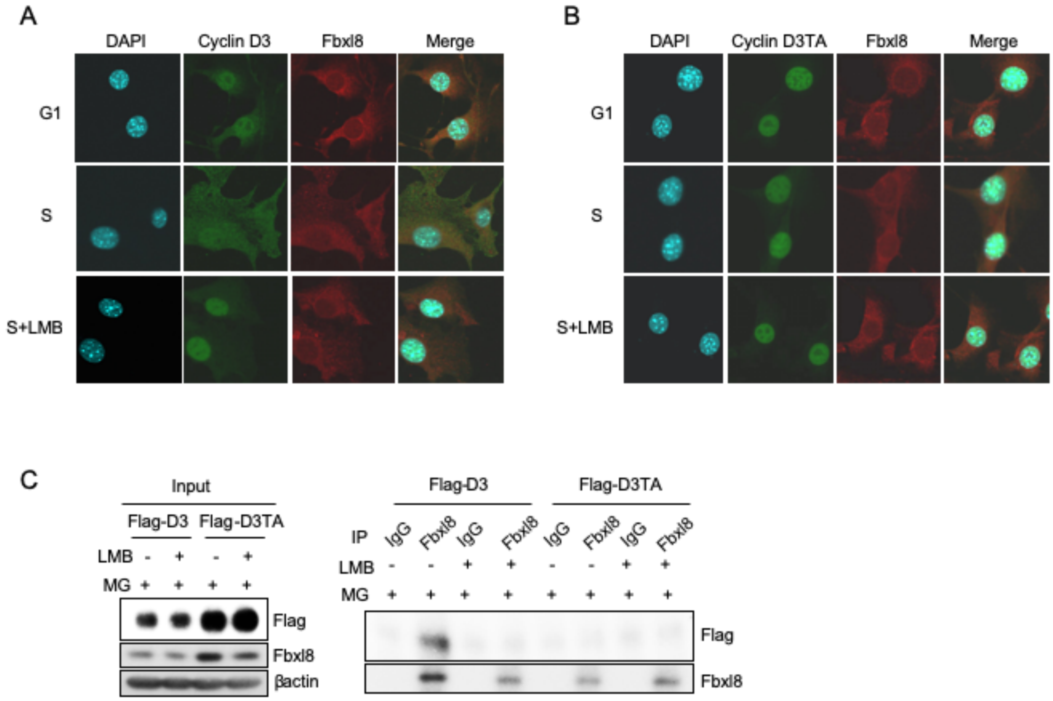

Figure S5. Subcellular localization of cyclin D3 during cell division.

(A, B) NIH3T3 cells were transfected with Flag tagged cyclin D3 (A) or Flag tagged cyclin D3T283A (cyclin D3TA) (B), and arrested at G0/G1 phase by serum starvation for 36 hours. Localization of cyclin D3 and Fbxl8 were assessed by immunofluorescence in cells at midG1 (8 hours) and S phase (16 hours) with or without treatment of Leptomycin B (LMB) (10 ng/ml), an inhibitor of nuclear export, for 2 hours after release from G0/G1 phase by re-splitting cells in DMEM with 10%FBS. Representative pictures were shown. (C) NIH3T3 cells were infected with Flag tagged cyclin D3 (Flag-D3) or Flag tagged cyclin D3T283A (Flag-D3TA), and arrested at G0/G1 phase by serum starvation for 36 hours. Binding of cyclin D3 or cyclin D3TA and Fbxl8 was assessed by immunoprecipitation using anti-Fblx8 antibody followed by western blot for Flag and Fbxl8 in cells at S phase (16 hours after release from G0/G1 phase) with or without treatment of Leptomycin B (LMB) (10 ng/ml) for 2 hours with a proteasome inhibitor MG132 (20 μM) for 4 hours.

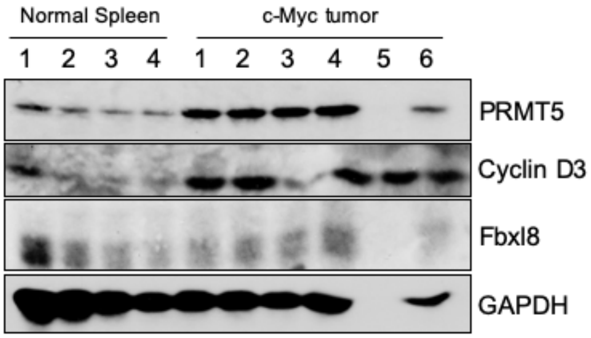

Figure S6. Cyclin D3 is overexpressed in c-Myc driven tumors.

Bone marrow cells isolated from 5-FU (150 mg/kg) treated mice were retrovirally transduced with MigR1/c-Myc in the presence of IL-3, IL-6, and stem cell factor (SCF) and then transplanted into lethally irradiated (900 rad) recipient mice. Lysates from c-Myc driven tumors and normal spleen cells were analyzed by western blot using antibodies against PRMT5, cyclin D3, Fbxl8 and GAPDH.

Yoshida_Fig. S7

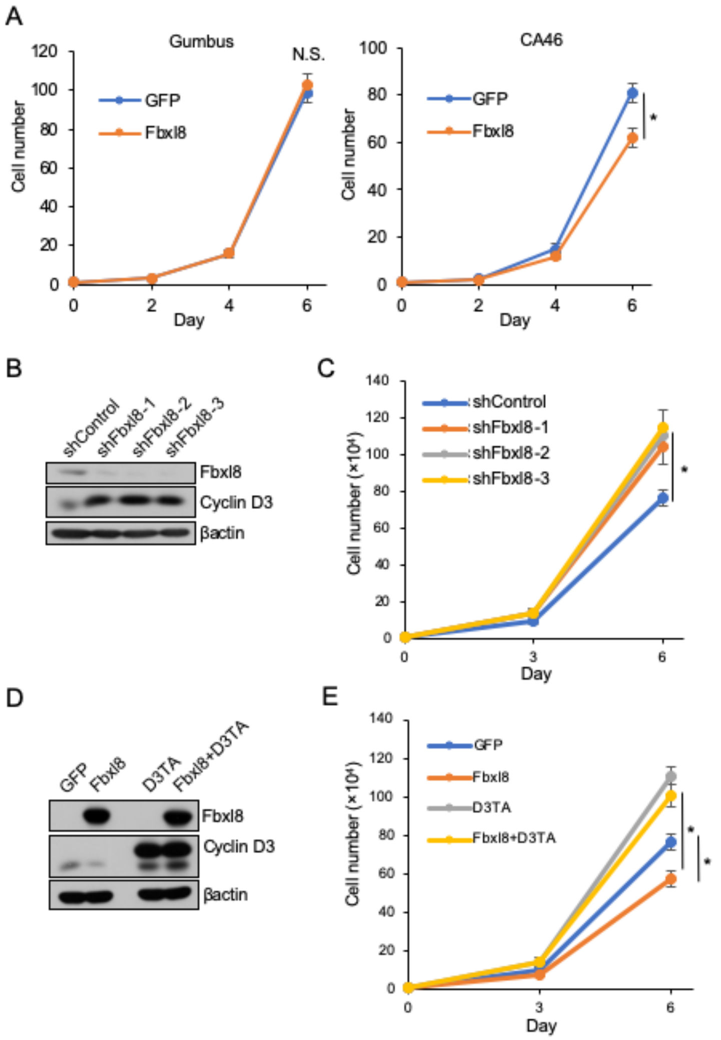

Figure S7. Fbxl8 regulates lymphoma cell proliferation through Cyclin D3.

(A) 1×10^4 Gumbus cells or CA46 cells infected with GFP or GFP/Flag-Fbxl8 were plated and cell numbers were counted every 2 days. Data represent mean ± SD, * $p<0.05$ (two-tailed Student's t-test, n=3). (B) Lysates from CA46 cells infected with shControl or shFbxl8s (1-3) were analyzed by western blot using antibodies against Fbxl8, cyclin D3 and βactin. (C) 1×10^4 CA46 cells from (B) were plated and cell numbers were counted every 3 days. Data represent mean ± SD, * $p<0.05$ (two-tailed Student's t-test, n=3). (D) Lysates from CA46 cells infected with GFP, Fbxl8, cyclin D3TA (D3TA), or Fbxl8+D3TA were analyzed by western blot using antibodies against Fbxl8, cyclin D3, and βactin. (E) 1×10^4 CA46 cells from (D) were plated and cell numbers were counted every 3 days. Data represent mean ± SD, * $p<0.05$ (two-tailed Student's t-test, n=3).

Yoshida_Fig. S8

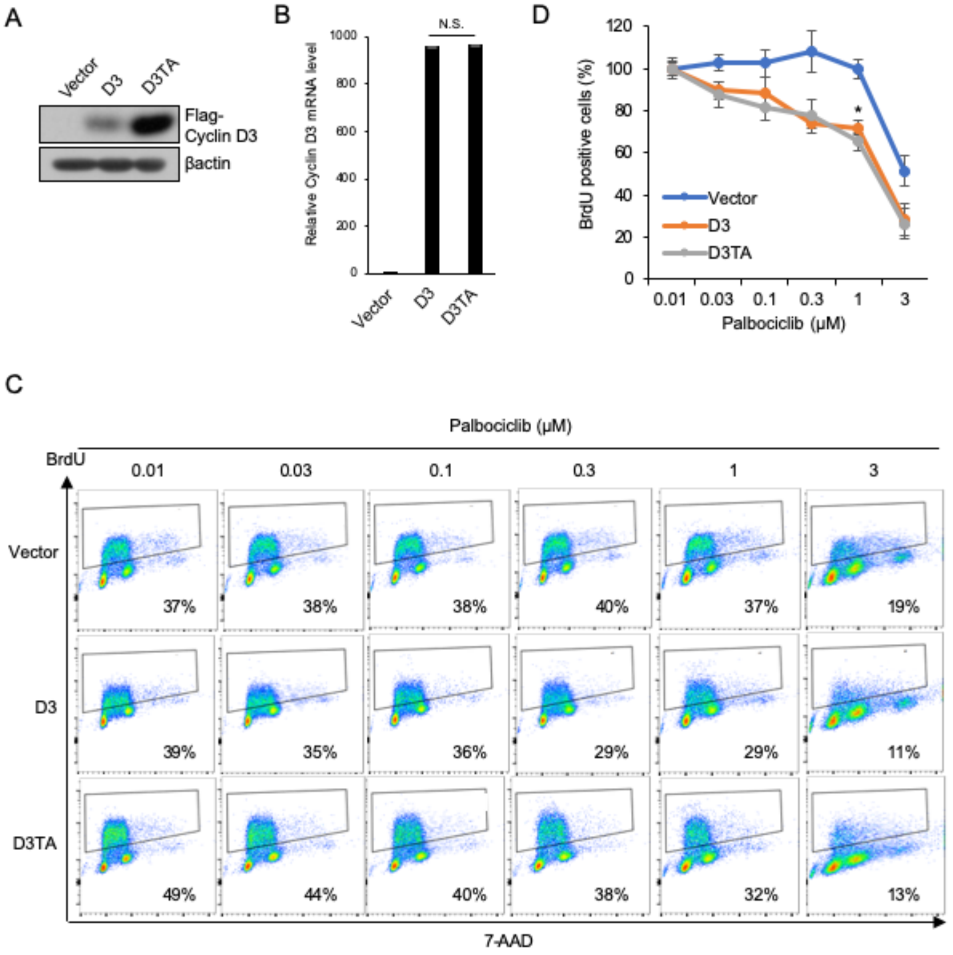

Figure S8. Cyclin D3 overexpression renders cells to susceptible to palbociclib.

(A) Lysates from NIH3T3 cells infected with vector, Flag-cyclin D3, or Flag-cyclin D3TA were analyzed by western blot using antibodies against Flag and βactin. (B) QPCR analysis of samples from (A) using a set of primer for cyclin D3. Data were normalized by GAPDH and represent mean ± SD. N.S., Not Significant (n=3). (C) NIH3T3 cells from (A) were subjected to BrdU incorporation for 40 min post palbociclib treatment for 2 days at different concentrations (0.01, 0.03, 0.1, 0.3, 1 and 3μM). BrdU and DNA content were determined by anti-BrdU (Y axis) and 7-AAD (X axis) respectively, and analyzed by FACS. (D) Quantification of BrdU positive cells from (C); mean ± SD, * $p<0.05$ (two-tailed Student's t-test, n=3).

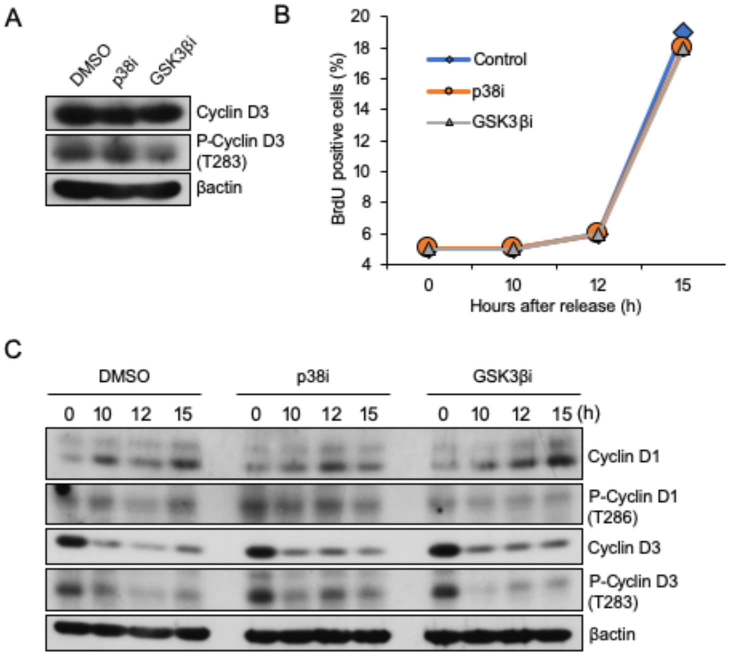

Figure S9. p38 or GSK3β marginally regulate cyclin D3 phosphorylation.

(A) Western analysis of lysates from CA46 cells treated with DMSO, SB203580 (p38 inhibitor) (10μM) or SB216763 (GSK3β inhibitor) (10μM) for 2 hours for phospho-cyclin D3, cyclin D3, cyclin D2 and βactin. (B) NIH3T3 cells were arrested at G0/G1 by contact inhibition for 36 hours. Following release by replating at low density, the S phase population was analyzed at 0, 10, 12 and 15 hours by BrdU incorporation assay. Quantification of BrdU positive cells were shown. DMSO, SB203580 (p38 inhibitor) (10μM) or SB216763 (GSK3β inhibitor) (10μM) were treated 2 hours prior to analysis. (C) Western analysis of lysates from (B) for phospho-cyclin D3 (T283), cyclin D3, phospho-cyclin D1 (T286), cyclin D1 and βactin.
